# Evidence for Decreased Brain Parenchymal Volume After Large Intracerebral Hemorrhages: a Potential Mechanism Limiting Intracranial Pressure Rises

**DOI:** 10.1007/s12975-017-0530-x

**Published:** 2017-03-09

**Authors:** Michael R. Williamson, Frederick Colbourne

**Affiliations:** 1 Neuroscience and Mental Health Institute, University of Alberta, Edmonton, Canada; 2 Department of Psychology, University of Alberta, P217 Biological Sciences Building, Edmonton, Alberta T6G 2E9 Canada

**Keywords:** Brain parenchymal volume, Intracerebral hemorrhage, Intracranial pressure, Pressure-volume relationship, Stroke

## Abstract

Potentially fatal intracranial pressure (ICP) rises commonly occur after large intracerebral hemorrhages (ICH). We monitored ICP after infusing 100–160 μL of autologous blood (vs. 0 μL control) into the striatum of rats in order to test the validity of this common model with regard to ICP elevations. Other endpoints included body temperature, behavioral impairment, lesion volume, and edema. Also, we evaluated hippocampal CA1 sector and somatosensory cortical neuron morphology to assess whether global ischemic injury occurred. Despite massive blood infusions, ICP only modestly increased (160 μL 10.8 ± 2.1 mmHg for <36 h vs. control 3.4 ± 0.5 mmHg), with little peri-hematoma edema at 3 days. Body temperature was not affected. Behavioral deficits and tissue loss were infusion volume-dependent. There was no histological evidence of hippocampal or cortical injury, indicating that cell death was confined to the hematoma and closely surrounding tissue. Surprisingly, the most severe hemorrhages significantly increased cell density (~15–20%) and reduced cell body size (~30%) in regions outside the injury site. Additionally, decreased cell size and increased density were observed after collagenase-induced ICH. Parenchymal volume is seemingly reduced after large ICH. Thus, in addition to well-known compliance mechanisms (e.g., displacement of cerebrospinal fluid and cerebral blood), reduced brain parenchymal volume appears to limit ICP rises in rodents with very large mass lesions.

## Introduction

After intracerebral hemorrhage (ICH), the mass effect from the hematoma and edema, among other factors, can raise intracranial pressure (ICP) [1]. High ICP predicts poor outcome and death [2, 3] and is linked to impaired consciousness [4]. However, ICP and related compensatory changes in response to large hemorrhagic mass lesions have not been well studied in animal models.

The Monro-Kellie hypothesis predicts that mobile cranial fluids, specifically blood and cerebrospinal fluid (CSF), are displaced to maintain ICP in response to a change in the volume of the cranial contents (e.g., from hemorrhage) [5, 6]. In contrast to the mutability of blood and CSF volumes, brain parenchymal volume, consisting of interstitial and intracellular fluids, is thought to be relatively unchangeable [5]. The exhaustion of rapid compensatory mechanisms (displacement of blood and/or CSF) due to a large insult causes ICP to increase, resulting in brain displacement or herniation and impaired cerebral blood flow [6–8].

We previously reported significantly (>20 mmHg) and persistently (at least 3 days) raised ICP following moderate and large collagenase-induced ICH in rats, with modest change after a lesion-size-matched whole blood-induced hemorrhage [9]. Here, we examined changes in ICP following striatal infusion of 100, 130, and 160 μL of blood. Most studies infuse 100 μL of blood or less in rats, which is comparable to a large ICH in patients, relative to brain size [9, 10]. We predicted that blood infusion would cause volume-dependent ICP elevations. In the collagenase model, ICP and edema peak 3 days after ICH [9]. Thus, we measured ICP for 72 h after ICH and then assessed brain water content. Further, most preclinical research measures brain water content in tissue containing the hematoma, but this may not reflect edema in surrounding tissue. Accordingly, we assessed whether increased brain water content following blood infusion was due to true peri-hematoma edema or to water contained within the hematoma. In addition, since temperature can impact ICP elevations [11] and large insults may impair temperature regulation, we measured whether it changed after large ICH. Also, we predicted that large hemorrhages would cause global ischemia as a result of high ICP [6]. We assessed this by histological examination of cells in the hippocampal CA1 sector and primary somatosensory cortex.

## Materials and Methods

### Subjects

Forty-seven male Sprague-Dawley rats (250–350 g, 8–12 weeks old) were obtained from the University of Alberta Biosciences colony. Histology from eight additional rats was used from our previous study [9], which was completed to the same standards as the present work. This study conforms to the RIGOR guidelines for translational research [12]. Animals were individually housed and had ad libitum access to food (Purina rodent chow) and water. Housing rooms were temperature and light controlled (lights on 7 AM to 7 PM). Group assignment was random and assessment performed blinded, except for behavioral assessment of the 160-μL-infusion group, which was added after assessment of the other groups. Group size was determined from a priori power analyses to achieve 80% power for major comparisons, based on results from pilot experiments and previous studies (mean ICP: *d* = 1.6, SD = 5.0 mmHg; lesion volume: *d* = 1.8, SD = 5.5 mmHg) [9, 11, 13].

### Experimental Protocols

**Fig. 1:**
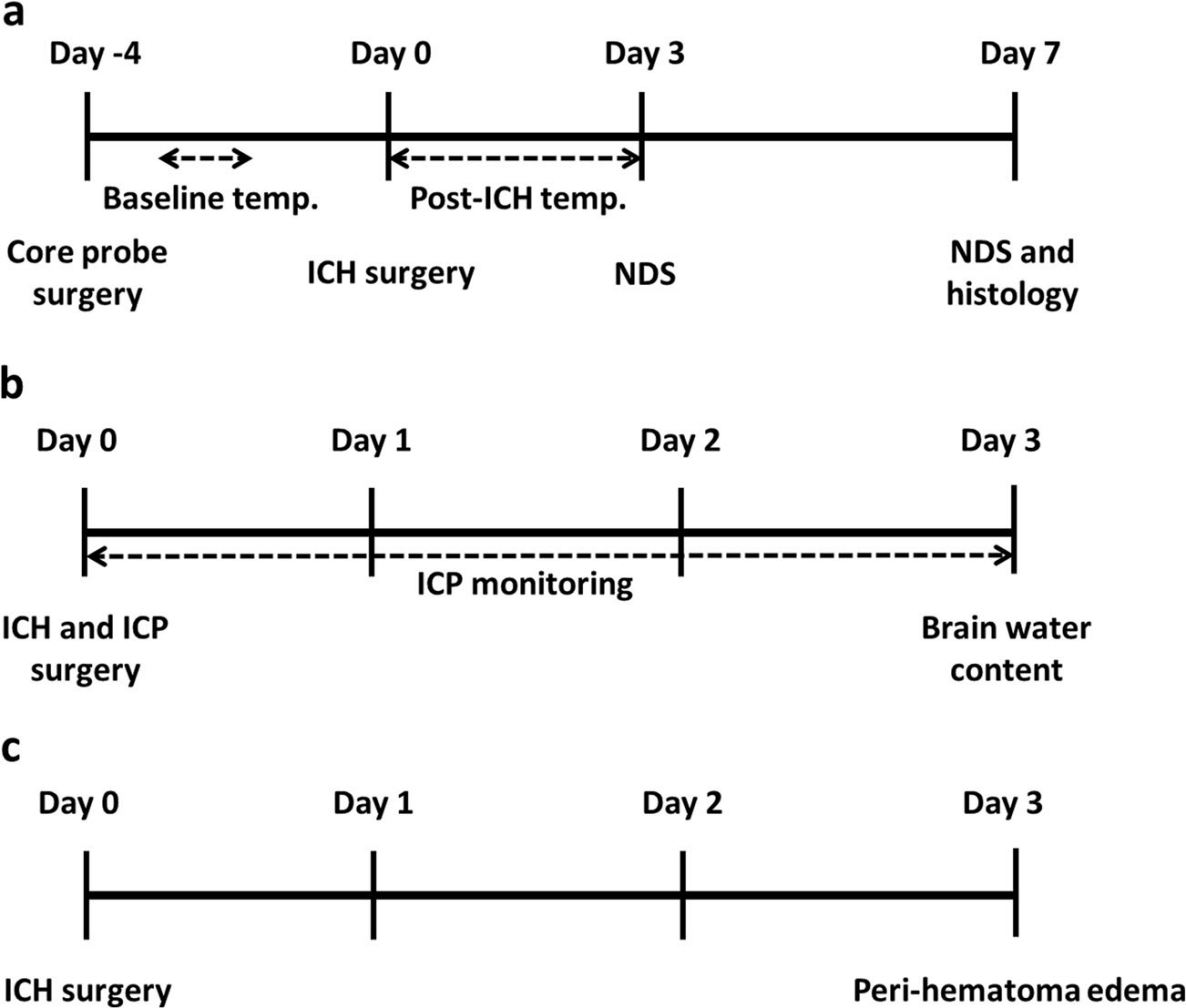
Experimental timelines. Timelines for experiments 1 (**a**), 2 (**b**), and 3 (**c**)

In the first experiment (Fig. 1a), we continually monitored core temperature and spontaneous movement after large striatal infusion of autologous blood. In addition, we measured cell size and density, lesion volume, and behavioral deficits. Five animals were used per ICH group, two for control. Tissue from previous work [9] with identical histological procedures was examined to compare cell size and density in the whole blood model with large collagenase-induced ICH (five ICH, three control). In the second experiment (Fig. 1b), we continually measured ICP for 3 days after ICH. Then, we assessed brain water content in post-mortem tissue (six rats per group). Lastly, in experiment 3 (Fig. 1c), we determined the extent of peri-hematoma edema in six animals subjected to striatal infusion of 130 μL of blood.

Surgical procedures were performed aseptically. Isoflurane (4% induction, 1.5–2% maintenance in 60% N_2_O, balance O_2_) was used for anesthesia. Marcaine (Sanofi Canada, Laval, QC) was used as a local anesthetic. Body temperature was continuously monitored during surgery with a rectal probe and was maintained at 37 °C with a heated water pad. Animals were monitored daily following surgery and given appropriate post-operative care (e.g., providing soft palatable food).

### Core Probe Implantation

Calibrated telemetry probes were used to measure temperature and activity in freely moving rats [14]. Probes (Model TA10TA-F40, Data Sciences Int., St. Paul, MN) were implanted 4 days before ICH surgery in the peritoneal cavity through a small incision. Data were recorded with Dataquest ART software (version 2.2, Data Sciences Int.). Measurements were recorded every minute and averaged every 30 min for analysis.

### Intracerebral Hemorrhage

Striatal hemorrhage was induced by infusion of autologous blood (i.e., the whole blood model) [9, 15]. A midline incision was made in the scalp and a burr hole was drilled 3.5 mm right and 0.5 mm anterior from Bregma. In animals receiving a pressure probe implant (described below), three additional burr holes were drilled surrounding the first to accommodate support screws. Blood was taken from the tail artery and 100, 130, or 160 μL was infused into the striatum (6.5 mm depth from the skull surface) through a 25-G needle over 10 min. The needle was removed in two steps over 13 min. Removal of the needle slowly over two steps eliminated backflow of blood up the needle tract that can occur during this procedure. No anticoagulant was used. Collagenase-induced ICH involved the infusion of 1 μL of 0.3 U of bacterial collagenase (Type IV-S, Sigma, Oakville, ON) and was done for our previous study [9]. The control procedure was identical except the needle was not inserted into the brain.

### Pressure Probe Implantation

Epidural ICP was continually measured using a method we developed [9, 13]. Immediately after ICH or control surgery, a telemetry transmitter (Model PA-C10, Data Sciences Int.) was connected to the epidural space above the infusion site. The probe was encased in a plastic cylinder that was secured to the skull with dental cement. Pressure was recorded every minute beginning ~90 min post-ICH (time to complete surgery and allow recovery) until 72 h post-infusion using Dataquest ART software (Data Sciences Int.). Artifacts presumably due to movement or electrical noise (>50 mmHg change in 1 min) were removed prior to analysis. Data were averaged over 30-min periods for analysis to reduce variability, except where otherwise noted. Pressure was adjusted for ambient pressure and corrected for offsets taken before implantation.

### Behavioral Testing

Animals that underwent behavioral testing were handled and tested prior to other procedures to reduce stress and confirm lack of presurgical impairment. Impairment was assessed using a Neurologic Deficit Scale (NDS) at days 3 and 7 post-ICH (experiment 1) [15]. Possible composite scores ranged from 0 (no deficits) to 14 (greatest deficits). Subtests evaluated forelimb flexion, hindlimb retraction, forepaw grasp, spontaneous circling, and beam walking ability.

### Brain Water Content

Animals were anesthetized and quickly decapitated. Brains were removed and blocked from 2 mm anterior to 4 mm posterior of the infusion site and separated into ipsilateral and contralateral hemispheres. The cerebellum served as a control. Tissue was weighed before and after baking (100 °C for 24 h). Water content was determined as a percentage of wet tissue weight [9].

In experiment 3, peri-hematoma edema was assessed in separate animals after a 130-μL infusion. Brains were removed and blocked and then separated into regions of interest as above. The hematoma was manually dissected from the ipsilateral hemisphere prior to determination of water content in peri-hematoma tissue. We were careful not to remove peri-hematoma tissue while removing the hematoma. Thus, a small amount of blood that was infiltrating residual tissue at the hematoma border was left in place.

### Histology

Animals from experiment 1 were given sodium pentobarbital (100 mg/kg, IP) and transcardially perfused with 0.9% saline and 10% neutral buffered formalin 7 days after ICH. Coronal brain sections (40 μm) were taken every 200 μm (~4.8 mm anterior to 4.8 mm posterior from the bregma) and stained with cresyl violet [14, 16]. Forty-micrometer sections are commonly used to assess injury area because there is less risk of tissue handling artifacts compared to thinner sections. Lesion volume was determined using ImageJ software (v. 1.48, NIH) as the area of the lesion multiplied by the distance between sections [15]. The lesion included injured tissue and intermixed hematoma discernible on cresyl violet-stained sections [9, 15]. Total volume of tissue loss was not assessed due to the significant distortion caused by large ICH. However, we note that this technique may not be as accurate to assess lesion size as measures done after hematoma resolution [15].

The same sections used to determine lesion volume were used to assess cell morphology in regions outside of the injured area. Pyramidal neurons were counted bilaterally in regions distal (rostral hippocampal CA1 sector, ~4.2 mm posterior to the bregma) and proximal (primary somatosensory cortex (S1), layer II/III, ~0.5 mm anterior to the bregma) to the hematoma (×40 objective, Olympus BX51-P; one section per region per animal) similar to previous work [15]. Neurons were confirmed to be healthy and centered within the sectioning plane by having intact cell walls and visible nuclei. Cells were counted within an unbiased counting frame [17] in medial, middle, and lateral regions of rostral CA1 and averaged across regions as commonly done [18]. Cell counts were also averaged from three random regions within S1. There was no evidence of cell death (e.g., karyorrhectic debris or reduced cell numbers) in these regions and the time scale was too short for formation of new pyramidal cells, so neuronal density changes appear to be attributable to altered extracellular or neuropil volume [19]. In addition, cross-sectional area of somas in each region was measured directly using ImageJ [20]. A grid was overlaid on images and in-focus cells intersected by the grid were analyzed with a systematic random sampling method (moving from top left to bottom right of an image until the requisite number of cells was analyzed; “classic” method) [17, 20]. Area was measured in 15 random cells per region (CA1 or S1) per hemisphere per animal. Values were not different between hemispheres so data was averaged between hemispheres for analysis. The CA1 sector and primary somatosensory cortex were chosen because they are vulnerable to global ischemia [18], which may occur from raised ICP [6]. Also, analysis of two disparate regions allowed us to determine whether changes in cell density or size were dependent on distance from the hematoma. The hippocampus was not directly affected by the hematoma. Primary somatosensory cortex was closer to the hematoma, but we did not assess tissue in the needle path or any tissue infiltrated with blood. We chose not to assess peri-hematoma regions to avoid confounds of ongoing injury, cell death, and edema [21–23].

### Statistical Analysis

Data are presented as mean ± standard deviation (SD) except as noted. Data were analyzed with GraphPad Prism (v. 6.0, GraphPad Software Inc., La Jolla, CA). Kruskal-Wallis tests followed by Dunn’s tests were used to analyze non-parametric data. Data measured over time were analyzed with two-way ANOVA (group and time variables) followed by Tukey’s post hoc tests where appropriate. Linear regression quantified the relationship between peak ICP and brain water content. All other data were analyzed with one-way ANOVA and Tukey’s post hoc tests or unpaired *t* tests. Assumptions of equal variance were tested with Brown-Forsythe or *F* tests. Statistical significance was defined as *P* < 0.05.

## Results

There was no mortality.

### Temperature and Activity

**Fig. 2:**
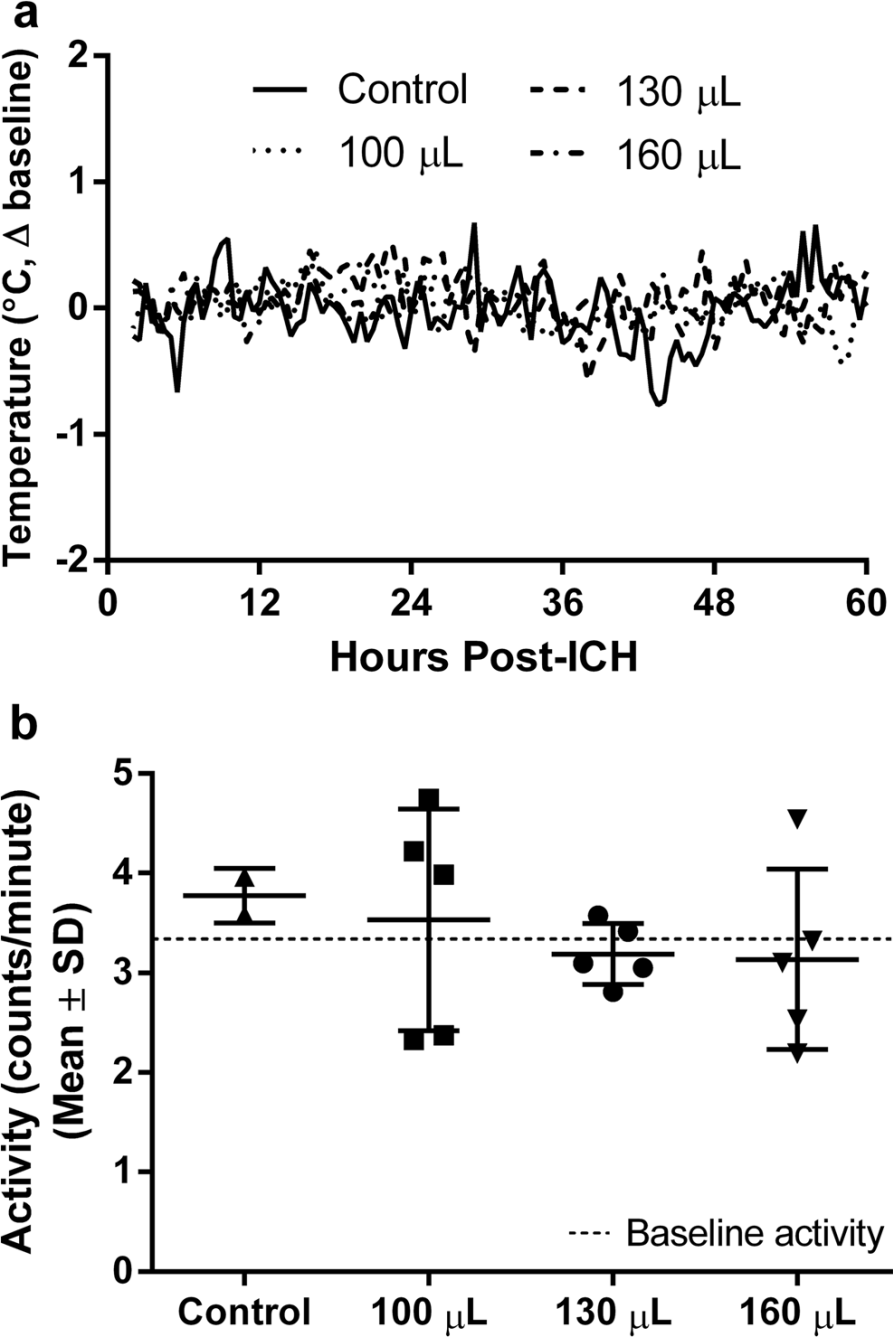
Temperature and activity after ICH. Temperature (**a**) and home cage activity (**b**) were not affected by ICH. The first 60 h of temperature data are presented as a difference from time-of-day-matched baseline temperature (positive values indicate hyperthermia). Activity data are presented as arbitrary counts per minute (measured by changes in signal strength due to movement). There were no baseline or post-ICH group differences. *N* = 5 rats per ICH group, 2 for control

One animal in the 130-μL-infusion group was excluded from analysis due to probe battery failure. Baseline (pre-ICH) temperature was the following: control 37.9 ± 0.3 °C, 100 μL 37.6 ± 0.2 °C, 130 μL 37.2 ± 0.4 °C, and 160 μL 37.3 ± 0.3 °C. Post-ICH temperature was the following: control 37.9 ± 0.3 °C, 100 μL 37.7 ± 0.3 °C, 130 μL 37.3 ± 0.3 °C, and 160 μL 37.4 ± 0.3 °C. The maximum range of temperature (averaged over 30-min epochs) was −1.0 to +1.3 °C from the individual average, which is natural variability due to the circadian rhythm. ICH surgery had no effect on temperature (*P* = 0.87, pre- vs. post-ICH). There were no group differences in temperature before or after ICH (Fig. 2a, *P* ≥ 0.34, ANOVA). Similarly, there were no average differences in home cage activity between groups before or after ICH (Fig. 2b, *P* ≥ 0.28, ANOVA).

### Lesion Volume and Impairment

**Fig. 3:**
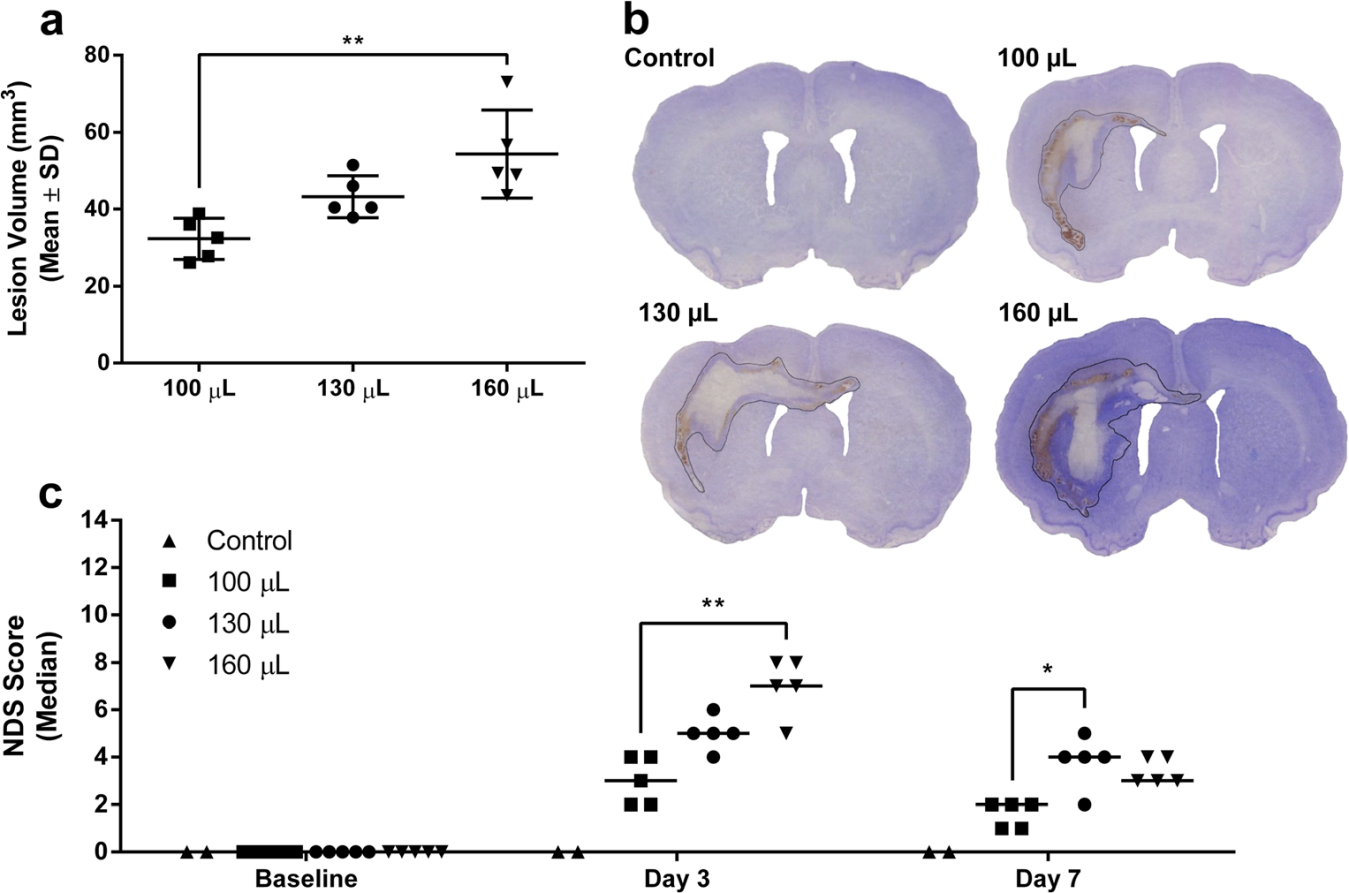
Lesion volume and behavioral impairment after ICH. Lesion volume (**a**; representative images are shown in **b**) and behavioral impairment (**c**) were infusion volume-dependent. *P < 0.05. **P < 0.01. *N* = 5 rats per ICH group, 2 for control. Control group was not included in analysis

Striatal blood infusion caused volume-dependent lesions (Fig. 3a, b; *P* = 0.003, ANOVA; *P* = 0.002, Tukey, 100 vs. 160 μL) that were largely confined to the striatum and corpus callosum, with some crossing to the contralateral hemisphere (2/5 100, 4/5 130, and 3/5 160-μL animals; <5% of the lesion extended to the contralateral hemisphere in all occurrences). There was no evidence of cerebellar herniation or intraventricular extension of the hematoma. Control animals had no lesion.

ICH caused severity-dependent behavioral impairments that were evident at 3 and 7 days post-ICH (Fig. 3c; *P* ≤ 0.003, ANOVA). Control animals were not impaired (score of 0).

### Intracranial Pressure

**Fig. 4:**
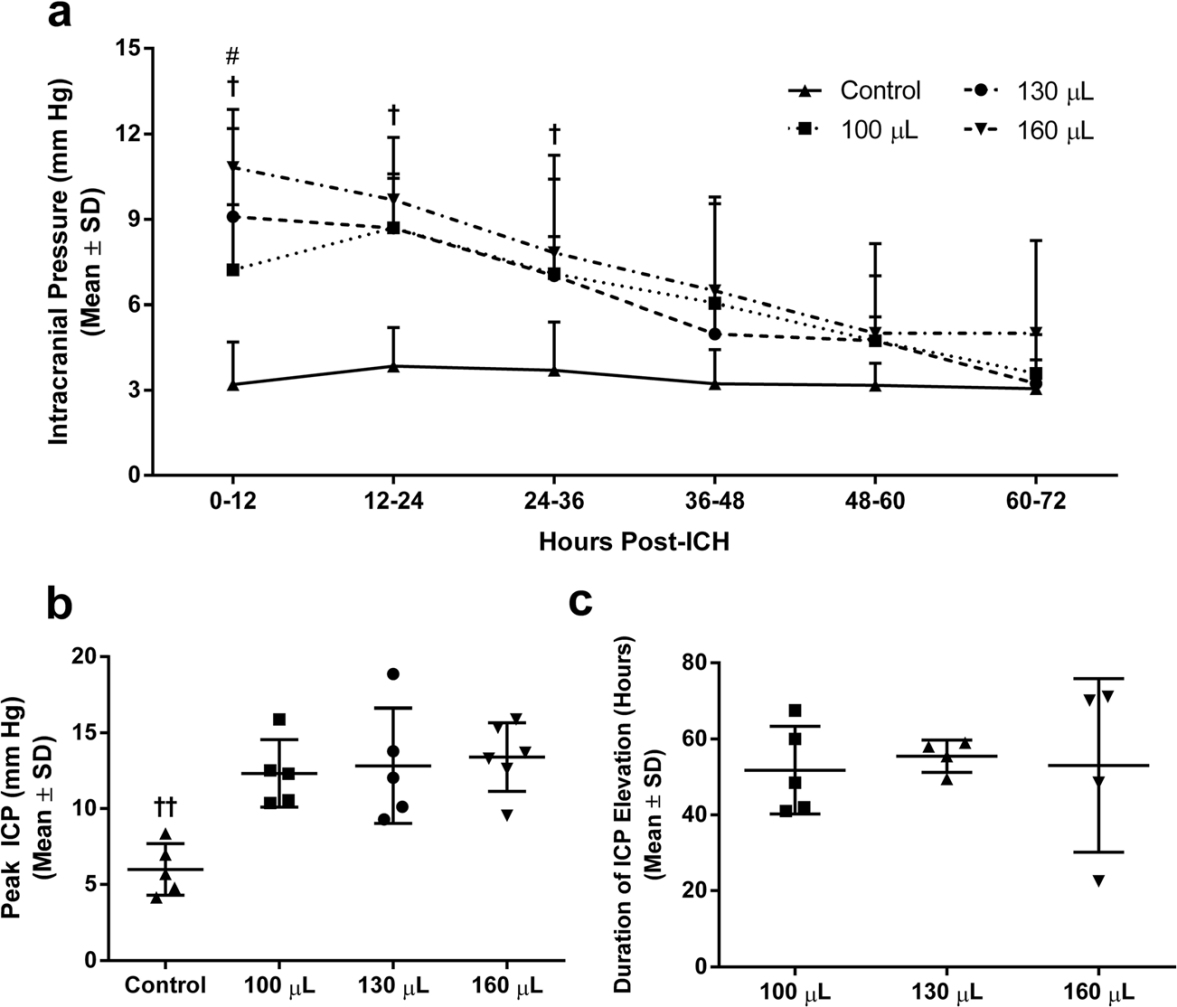
Intracranial pressure after ICH. Whole blood-induced ICH caused immediate, but modest, ICP rises that declined over time (**a**, mean ICP averaged over 12-h epochs). Control: *N* = 5, 100 μL: *N* = 5, 130 μL: *N* = 5, 160 μL: *N* = 6. Peak ICP (over 30 min) was not different between ICH groups (**b**). The duration of ICP elevations was not different between ICH groups (**c**). Duration was calculated as the total time during which 30-min ICP means were above the 95% confidence interval of the control group. ^†^ *P* < 0.05 ICH vs. control. ^††^ *P* < 0.01 ICH vs. control. ^#^ *P* < 0.05 between ICH groups (see “Results”)

One animal from each of the control, 100-μL-, and 130-μL-infusion groups was excluded from ICP analysis due to catheter blockage. One animal from the 160-μL-infusion group had data collected over only the first 30 h before probe battery failure. One additional animal from each of the 130- and 160-μL groups was excluded from duration analysis due to loss of signal for >30 min (e.g., from excess distance between probe and receiver). Intracerebral hemorrhage caused a transient elevation in ICP in all groups followed by a gradual return to control levels (control 3.4 ± 0.5 mmHg; Fig. 4a). Intracranial pressure was elevated above control immediately following ICH, regardless of hematoma size (Fig. 4a; *P* ≤ 0.03, Tukey, ICH vs. control). During the first 12 h, ICP in the 160-μL group was significantly higher than that in the 100-μL ICH group (*P* = 0.045, Tukey). From 0 to 36 h, ICP in at least one ICH group was greater than that in the control (*P* ≤ 0.02, Tukey, ICH vs. control; all ICH groups were greater for the first 24 h, only 160 μL for 24–36 h). We found no group differences in peak ICP (the highest ICP averaged over a 30-min period; Fig. 4b; *P* ≥ 0.90, Tukey) or duration of the pressure elevation (Fig. 4c; *P* = 0.93, Tukey) among ICH groups.

### Brain Water Content

**Fig. 5:**
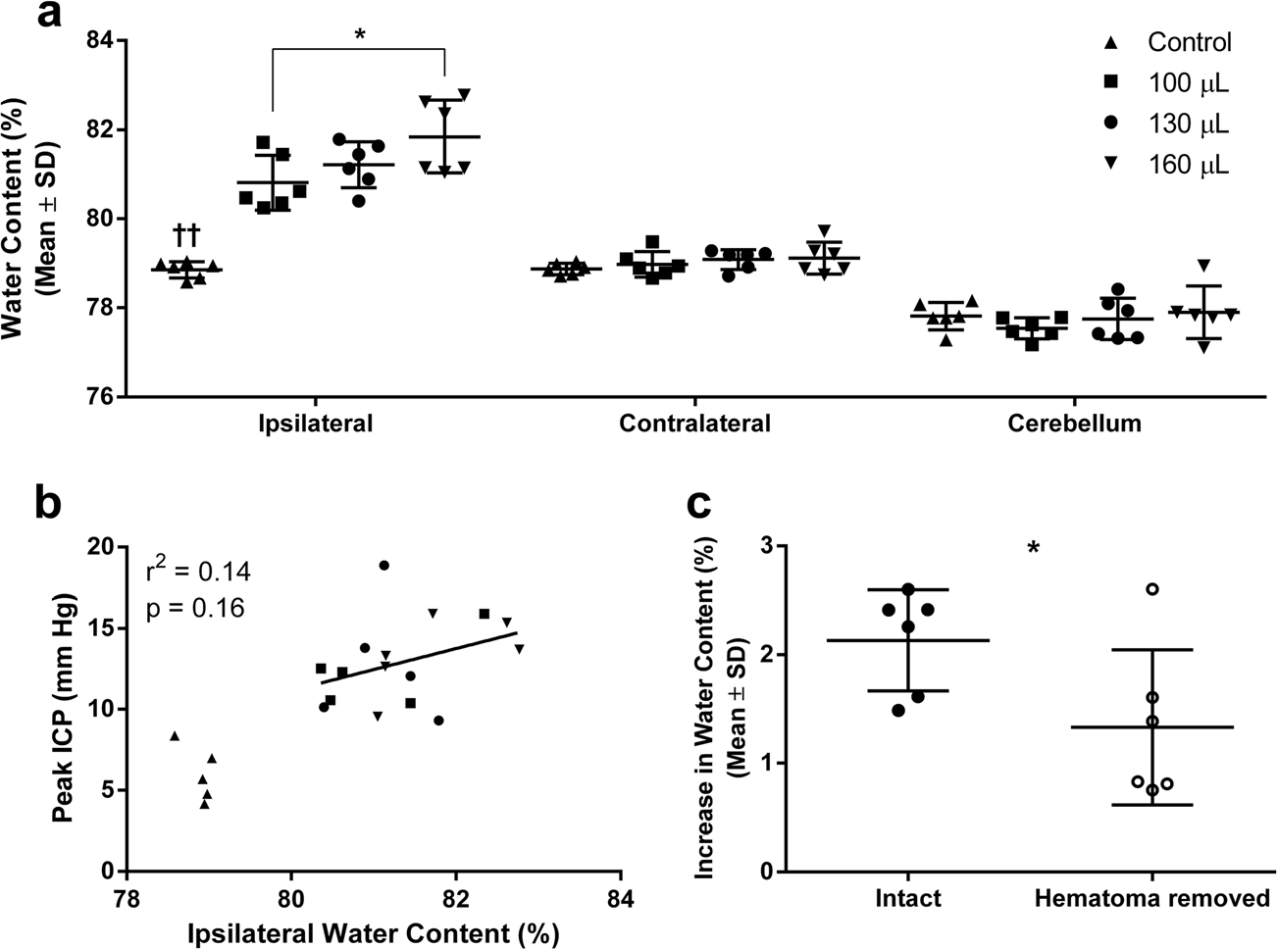
Edema after ICH. Brain water content in the ipsilateral hemisphere was increased after ICH in an infusion-volume-dependent manner (**a**). Linear regression between ipsilateral hemisphere water content and peak ICP revealed no significant relationship (**b**). Regression includes ICH groups only. There is modest peri-hematoma edema after blood infusion; brain water content measurements are mostly due to water contained within the hematoma in this model (**c**). *P < 0.05. ^††^ *P* < 0.01 ICH vs. control. *N* = 6 rats per group, unless otherwise specified

Water content in the ipsilateral hemisphere was infusion-volume-dependent and greater after ICH than control surgery regardless of infusion volume (Fig. 5a; *P* < 0.001, Tukey, ICH vs. control). As expected, there were no significant group differences in the contralateral hemisphere or cerebellum (*P* ≥ 0.39, ANOVA), which served as controls. There was no significant relationship between ipsilateral water content and peak ICP after ICH (Fig. 5b; *P* = 0.16, *r* ^2^ = 0.14). As we previously found [9, 11], the relationship was significant when control animals were included in the regression (*P* < 0.001, *r* ^2^ = 0.59), but including control animals does not assess the relationship between water content and ICP after ICH.

Water content in brains with the hematoma removed (i.e., peri-hematoma edema) was compared with the water content of the 130-μL (hematoma intact) group in experiment 2. Data are presented as an increase from the contralateral hemisphere because of a small, but statistically significant, group difference in contralateral water content. Peri-hematoma water content was significantly less than in hematoma-containing tissue (Fig. 5c; *P* = 0.02). Sampling of tissue containing the hematoma is routinely done. Thus, a significant amount of the increased water content reported for this model is due to hematoma in the sample.

### Cell Size and Density

**Fig. 6:**
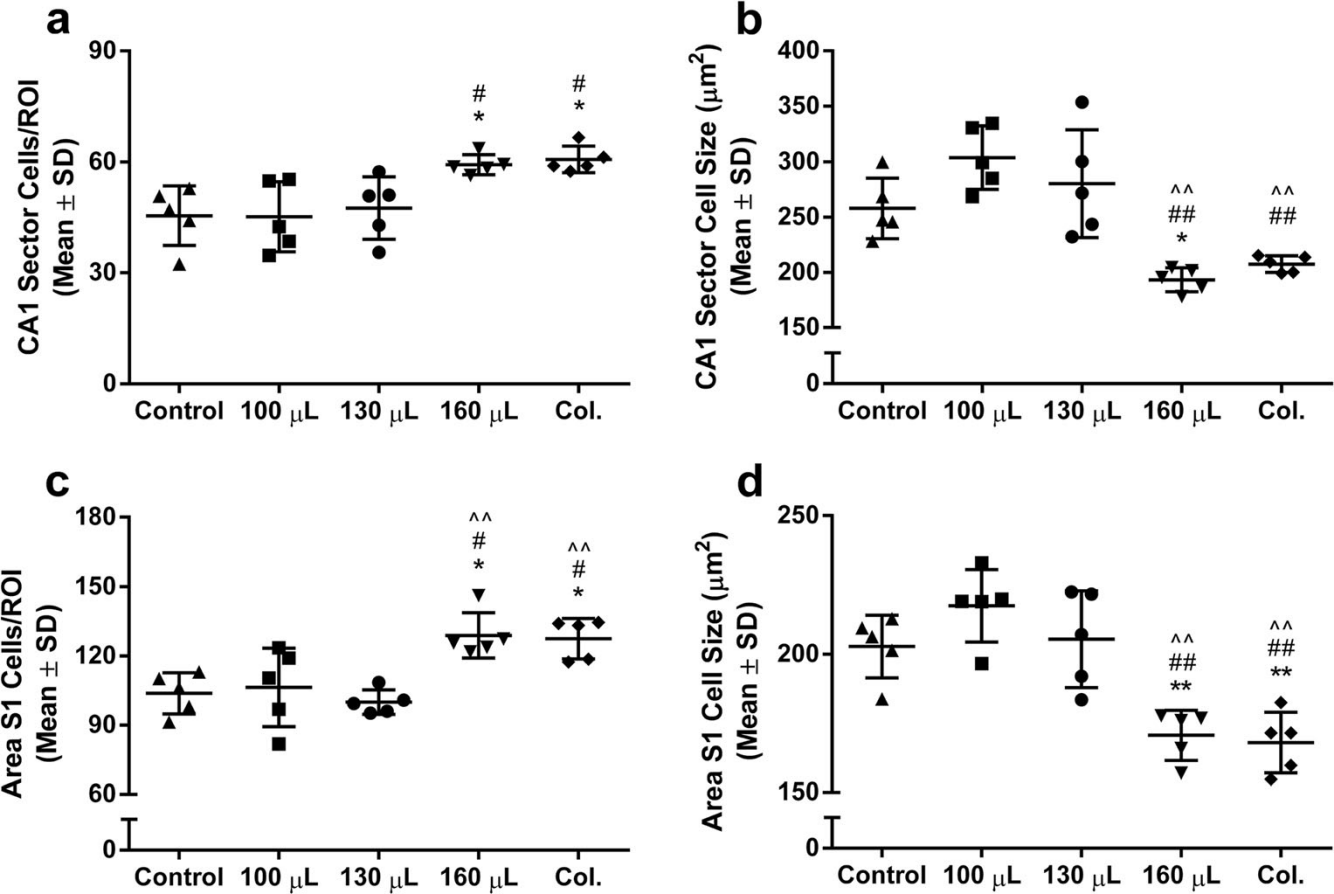
Cell size and packing density after ICH. Very large ICH induced by 160 μL of autologous blood or 0.3 U collagenase (*Col.*) [9] increased cell-packing density and decreased cell size in the rostral hippocampal CA1 sector (**a**, **b**) and primary somatosensory cortex (**c**, **d**). Effects were bilateral. Smaller ICHs did not affect cell size or density. Cell density is reported as the average number of cells within a 250 × 250 μm region of interest (*ROI*) corresponding to the size of the microscope grid reticle through a ×40 objective. *P < 0.05, ** *P* < 0.01 vs. control. ^#^ *P* < 0.05, ^##^ *P* < 0.01 vs. 100 μL. ^^^ *P* < 0.01 vs. 130 μL. *N* = 5 per group

**Fig. 7:**
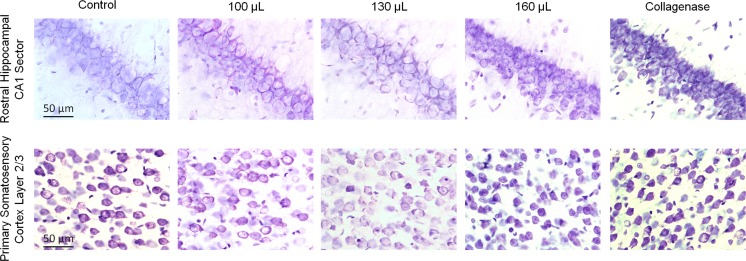
Representative images of cresyl violet-stained tissue demonstrate decreased cell size and increased cell density in the hippocampal CA1 sector and primary somatosensory cortex

We assessed cell size and density bilaterally in the hippocampal CA1 sector and S1 cortex. Very large hemorrhages induced by whole blood (160 μL) or collagenase (0.3 U) decreased cell size (by ~30%) and increased packing density (by ~15–20%) relative to smaller hemorrhages and/or control in both areas (Figs. 6 and 7; *P* ≤ 0.002, ANOVA, see Fig. 6 for specific comparisons). These effects are not attributable to cell death because there was no morphological evidence of injured or dead cells in the areas assessed. There were no differences between ipsilateral and contralateral hemispheres for cell density (*P ≥* 0.45) or cell size measurements (*P* ≥ 0.58).

Dehydration may confound histological analysis [24] but does not appear to be a factor here because weight recovery from time of ICH or control surgery to euthanasia was equal in all groups (*P* = 0.069, ANOVA; average weight gain over the 7 days was as follows: control 63 ± 7 g, 100 μL 34 ± 20 g, 130 μL 30 ± 7 g, 160 μL 33 ± 12 g) and brain water content in control structures was unchanged (refer to previous sub-section).

## Discussion

Raised ICP is a potentially deadly consequence of ICH. A thorough understanding of pressure rises and compensatory mechanisms is vital to developing novel treatments. However, studying these concepts requires appropriate animal models. Here, we show that large hemorrhages induced by striatal blood infusion are readily accommodated for, causing ICP to increase only modestly and transiently. Thus, at least with regard to ICP, this particular model does not accurately mimic severe ICH in patients. Interestingly, we found that very large mass lesions in both common rodent models of ICH caused cell density to increase and cell size to decrease in the hippocampus and cortex, and likely in other brain regions. These initial findings may challenge the long-held notion that the brain is largely incompressible because large mass lesions appear to compress parenchymal volume.

The transient nature of the ICP rises reflects compensatory changes in the volumes of cranial contents to preserve normal pressure [5, 6]. The conventional view of cranial pressure-volume relationships describes three cranial volumes: the blood, CSF, and brain. However, despite occupying >80% of the cranial space, brain parenchyma has not been considered a mutable cranial volume, in contrast to the other rapidly displaced cranial fluids [25]. Our findings that cell-packing density is increased bilaterally in two disparate brain regions after large ICH suggest that interstitial fluid is displaced or neuropil volume is reduced. Similarly, decreased cellular size indicates a decrease in intracellular fluid. Thus, large mass lesions can seemingly displace parenchymal volume, an unexpected finding of potential therapeutic interest.

We show evidence of decreased brain volume in two ICH models. The whole blood model produces a much larger hematoma than a lesion size-matched collagenase insult; however, the collagenase model causes much more edema [11, 15]. Therefore, the mass effect of each is significant. Notably, effects were bilateral, consistent with our previous findings that there are no easily detectable pressure gradients after rodent ICH [9]. We also note that decreased cell size and increased density were observed only after the most severe hemorrhages, indicating that easily displaced fluids are moved first. This implies that parenchymal volume can be diminished outside the hemorrhagic zone. Furthermore, this decreased volume is not attributable to cell death or sub-lethal injury because we found no histological evidence of global ischemia (e.g., cellular debris and greater microglia numbers [26]) and we assessed regions outside of the pathologically active peri-hematoma zone. Moreover, these changes are not likely due to the inflammatory response, which tends to be localized to the hemorrhage site and is resolving by day 7 [27]. As well, infiltration of inflammatory cells would presumably increase parenchymal volume, which is the opposite of what we observed in the cortex and hippocampus. We assessed changes in cell size and density 7 days after ICH, which means the observed changes in cell size and density are persistent. However, it is unclear when this shrinkage begins, and it may have occurred after smaller insults but resolved before 7 days. We speculate that mechanical force due to the mass lesion (i.e., the mass effect) drives an efflux of interstitial fluid from the brain along paravascular routes [28], though this should be tested directly. It remains unclear if cellular shrinkage is an active (regulated [29]) or passive (mechanical [28]) process.

Direct assessment of the implications of decreased cell size and increased density was beyond the scope of this study. Nonetheless, it should be noted that stroke impairs metabolism and function of surviving tissue [30] and high ICP depresses consciousness [4]. Further, changes in interstitial volume are associated with large-scale state-dependent activity changes [29], and changes in cellular volume can alter excitability [31]. Notably, seizures occur after collagenase-induced ICH, but not after a 100-μL blood infusion [16]. Seizure activity may occur in very large blood-induced hemorrhages that cause changes in cell size and density. Taken together, these findings suggest that changes in intracellular and interstitial volume may contribute to altered neural activity after stroke, though this should be tested directly.

We previously reported ICP elevations above 20 mmHg following collagenase-induced ICH [9, 11]. Here, we found severity-dependent, but modest (<20 mmHg), increases in ICP after large ICH in the whole blood model. Further, the temporal pattern of the pressure response differs between models. The collagenase model raises ICP for at least 3 days after hemorrhage, whereas blood infusion causes immediate, but short-lived, increases. It is possible that ICP increases over time in some models due to the progression and expansion of the lesion and edema, which occurs to a much greater extent in the collagenase model [15]. Notably, delayed ICP peaks are also observed in models of focal ischemia [13, 32].

Edema is a common endpoint in preclinical ICH research [33]. While edema is thought to be a major contributor to raised ICP [1], recent studies show a complex relationship [11, 32, 34]. Here, we found that much of the increase in brain water content after ICH was due to water contained within the hematoma. This suggests that there is relatively little peri-hematoma edema in the whole blood model (vs. collagenase model), even with massive hematoma size. However, it is possible that the 160-μL infusion caused more peri-hematoma edema than the smaller infusions. In addition, we found no significant relationship between brain water content and peak ICP in ICH animals. Taken together, these findings suggest a limited role of edema in ICP rises in this model; the hematoma itself is the primary source of the mass effect in some cases. This hypothesis is consistent with clinical findings that peri-hematoma edema appears to be significantly derived from hematoma-constrained water [35]. Furthermore, the relationship between edema and ICP is highlighted by treatment effects. We [11] and others [32, 34] have reduced post-stroke ICP elevations using hypothermia. In these studies, ICP was lowered without affecting edema. While these findings conflict with evidence that hypothermia reduces edema [36], they support the notion that edema is not alone in causing ICP elevations after brain injury. While edema may reflect outcome, it may not predict ICP changes as previously thought and appears to be commonly overestimated due to including hematoma in samples. Of course, edema measurement with the wet-dry weight method is dependent on the tissue sample size (e.g., extent of normal and abnormal tissue), making cross-study and cross-technique comparisons challenging [37]. Thus, the use of ICP measurement, although technically challenging in rodents, is recommended when done with an established and consistent method. Additional measures, such as tissue oxygenation, may better predict outcome when used alone or in conjunction with ICP recordings [38].

Preclinical investigation of ICP has produced considerable inter-study variability in the absolute magnitude of ICP measurements, even in uninjured animals. For example, one study reported resting ICP in rats from 17 studies as ranging from 0 to 47 mmHg [39]. Most reports indicate that normal rat ICP is 4 to 8 mmHg, compared to ~10 mmHg in humans [6, 39]. In the present study, we observed ICP in uninjured animals to be 3.4 ± 0.5 mmHg, which is slightly lower than our past studies likely due to normal variation within the population (i.e., sampling chance) or some unknown technical issue. We previously found ICP in awake control animals to be ~4 to 5 mmHg using this technique [9, 11, 13] and with non-telemetric, fluid-filled transducers in anesthetized animals (unpublished data). Nonetheless, the lack of consistent measures between groups and studies is concerning. Differences in technique and location may underlie the variability, though the most common recording sites (intraventricular, intraparenchymal, and epidural) seem to produce the same measurements within studies [9, 39, 40], but measurement from the cisterna magna may underestimate ICP [40]. We chose epidural measurement due to the ease of access and lack of tissue damage compared to other common sites. Greater inter-study consistency in methodology would ease interpretation and comparison of findings.

There are several limitations with our methodology. First, there is a delay between ICH and the beginning of ICP recordings. Since we tend to observe the highest ICP soon after ICH in the whole blood model, we may have missed important early spikes. However, it takes time for ICP to normalize following opening of the cranium for blood infusion. Nonetheless, ICP does not increase enough to significantly reduce cerebral blood flow because there was no evidence of widespread global ischemia (i.e., no CA1 sector cell death, no spikes in ICP to levels which would significantly impair cerebral blood flow), though we did not assess whether localized peri-hematoma hypoperfusion or hypoxia occurred [6]. Second, we were not able to correlate behavioral impairment and lesion volume with ICP because these outcomes were measured in separate animals. However, we observed infusion-volume-dependent changes in mean ICP, lesion volume, and impairment between experiments. Future studies should directly assess the relationship between ICP, related mechanisms (e.g., tissue oxygenation), and histological and behavioral outcomes in preclinical models. Also, it is unlikely that fixation artifacts confound our findings on cell size and density because all tissues were treated identically. Nissl stains tend to underestimate cell volume due to restriction to the Nissl body and tissue shrinkage, but underestimates are linear [41, 42]. Thus, our comparisons between identically processed samples are seemingly valid, but we acknowledge that our measurements of soma size may overrepresent larger cells [20]. Lastly, we did not assess total parenchymal volume changes because that was not our initial aim, and changes were assessed at only one time post-ICH. Future studies could chronically assess brain volume using MRI, but mass effects may confound these measures. Alternatively, interstitial and cellular volumes could be assessed by other methods, such as iontophoresis and stereology, respectively. We did not assess total or regional parenchymal volumes because our cell density and size findings were post hoc findings and we only took enough sections to assess lesion volume. More sophisticated stereological assessment may provide more accurate measures of cell size and density [17, 20]. Thus, future studies are essential to test and extend our initial findings and interpretation.

The threshold at which ICP rises become dangerous should be clarified. Well-defined thresholds for harmful ICP would aid clinical management [43]. Currently, a thorough understanding of ICP thresholds is lacking, especially for brain hemorrhage. Indeed, current guidelines suggesting a threshold of 20 mmHg are largely based upon management principles for traumatic brain injury [1]. Notably, we observed ICP >20 mmHg to be potentially fatal in rodent stroke models [9, 13]. Alternatively, future studies could directly assess cranial compliance after brain injury. The ability of the cranial space to compensate for pressure changes may better relate to outcome than ICP alone because of the predictive potential of compliance measures (e.g., pressure-volume index) for dangerous pressure spikes [43]. However, it is unclear how predictive rat ICP changes are of the human case or even how best to analyze ICP [44]. We also note that studies on the benefits of ICP monitoring after acute brain injury are inconclusive [45].

In summary, we show that severe ICH induced by large striatal infusion of blood does not cause significant and persistent ICP rises, indicating that the whole blood model of ICH does not adequately mimic the human condition in this regard. In addition, we provide initial evidence that brain parenchymal volume markedly decreases in response to large hemorrhagic lesions in the autologous blood infusion and collagenase models. Diminished parenchymal volume coupled with little peri-hematoma edema is perhaps the most parsimonious explanation as to why the blood infusion model does not cause large ICP rises, but this should be confirmed. These findings may question the doctrine that CSF and blood are the only sources of compensatory reserve to maintain normal ICP. The functional impact of reduced parenchymal volume should be assessed. We encourage further study on the mechanisms underlying ICP rises and compensatory responses after brain injury so that more effective treatments can be developed.
